# Field Correlations in Surface Plasmon Speckle

**DOI:** 10.1038/s41598-019-44780-5

**Published:** 2019-06-07

**Authors:** Matthew R. Foreman

**Affiliations:** Blackett Laboratory, Imperial College London, Prince Consort Road, London, SW7 2AZ United Kingdom

**Keywords:** Nanophotonics and plasmonics, Nanophotonics and plasmonics

## Abstract

In this work fluctuations in the electric field of surface plasmon polaritons undergoing random scattering on a rough metallic surface are considered. A rigorous closed form analytic expression is derived describing second order correlations in the resulting plasmon speckle pattern assuming statistically stationary and isotropic roughness. Partially coherent planar Schell-model source fields can also be described within the developed framework. Behaviour of the three-dimensional degree of cross polarisation and spectral degree of coherence is also discussed. Expressions derived take full account of dissipation in the metal with non-universal behaviour exhibited within the correlation length of the surface and source fields.

## Introduction

Wave propagation in disordered media can give rise to a variety of interesting physical phenomena such as Anderson localisation and coherent back scattering^1–3^. Ultimately such coherent effects derive from phase correlations which persist between elementary scattered waves in spite of the randomisation imparted by the media. Intensity correlations are also known to exist in the random interference pattern, or speckle, produced by wave scattering in such media. For example, short range intensity correlations, often denoted *C*_1_, give rise to the characteristic size of an individual speckle^4,5^, whereas in the multiple scattering regime longer range *C*_2_ and *C*_3_ correlations are responsible for enhanced mesoscopic fluctuations^6^ and deviations from standard Rayleigh statistics^7,8^. An infinite range space-time correlation, *C*_0_, has also been predicted in speckle patterns produced by point sources embedded in disordered media^9^. Developments in the theoretical understanding of such spatial and temporal correlations of light in scattering media^3,6^ have in turn enabled a number of novel experimental techniques in, for example, imaging^10^, particle analysis^11^ and wave control^12,13^. Recently it has furthermore been recognised that evanescent contributions to the field inside a random medium, or at sub-wavelength distances from its exit surface, can significantly modify the properties of speckle patterns, by introducing sub-wavelength correlations^14,15^ and a polarisation dependence^16–18^. Non-universal behaviour^19,20^, whereby the statistical properties of the scattered field are strongly dependent on the statistics of the surface, can also occur in contrast to far-field speckle^21,22^.

Resonant surface waves, such as surface exciton-polaritons^23^, surface phonon-polaritons^24^, or surface plasmon-polaritons (SPPs)^25^, can also play an important role in scattering at interfaces and thus can affect the physical properties of random electromagnetic fields. Spectral variations with distance from a surface can, for instance, arise due to the characteristic exponential fall off of surface phonon polaritons in silicon carbide^26^, whereas SPP scattering can give rise to a flat absorption band in the infra-red part of the spectrum when supported in random metallo-dielectric films close to the percolation threshold^27^. Spatial correlations in the near field speckle intensity have also been considered in a number of studies, partly due to their experimental accessibility. Both oscillatory and monotonically decaying intensity correlation functions have for instance been observed near semi-continuous metal interfaces, where the dominant behaviour is dictated by the degree of SPP scattering^28^. Signatures of random SPP scattering can also be exhibited in far-field intensity correlations^29^. Intensity correlations however do not capture lower order statistical properties such as correlations between different field components. Field-field correlations, as parametrised by the cross spectral density matrix^30^, have thus also seen attention in the literature and capture both coherence and polarisation properties of randomly scattered fields. Strong near field polarisation effects in the presence of SPPs or surface phonons have for example been predicted^31,32^, whereas surface waves excited by thermal emission from a half space have been shown to give rise to long coherence lengths^33^. Anisotropy in the coherence length has also been analysed^34^. Notably, the cross-spectral density matrix can also be related to the density of states in disordered systems^35,36^, which is of fundamental importance since it governs many light matter interactions, such as fluorescence and thermal emission. Fluctuations in the local density of states near rough and fractal metallic films, resulting from random interference of SPPs, have been experimentally observed and linked to SPP localisation^37,38^.

In this work we consider the field correlations present in the random scattering of surface plasmon polaritons (SPPs) propagating on a rough surface in closer detail. Specifically, we provide fully analytic closed-form expressions for correlations, present in the roughness induced field fluctuations in SPP speckle (Eqs (9–11)). Our derivation exploits the known analytic form of the Green’s tensor describing SPP scattering^39^. These formulae help generalise and validate the predications of more specialised and approximate treatments given in the literature, which for example either assume the exciting source or surface correlation function is well described by a Dirac delta function (e.g. by considering a thermal source), or factor out slowly varying terms from the full integral representation of the cross-spectral density matrix^31–33^. Our formulae therefore afford greater physical insight by enabling description of more general surface correlation functions where the only restriction made is that of isotropic stationary statistics. Due to the complexity of the formulae we restrict attention to the single scattering regime, such that we can employ the well known equivalent surface current model for scattering from random metal interfaces as initially described by Kröger and Kretschmann^40,41^. We conclude by using our formulae to study the spatial polarisation and coherence properties of SPP speckle. The former is possible since our derivation yields the full correlation matrix, including off-diagonal terms. Such properties can be relevant in, for example, plasmonic interferometry^42,43^, plasmonic focusing^44^ and leakage radiation microscopy^45,46^.

## Theoretical Derivations

**Figure 1:**
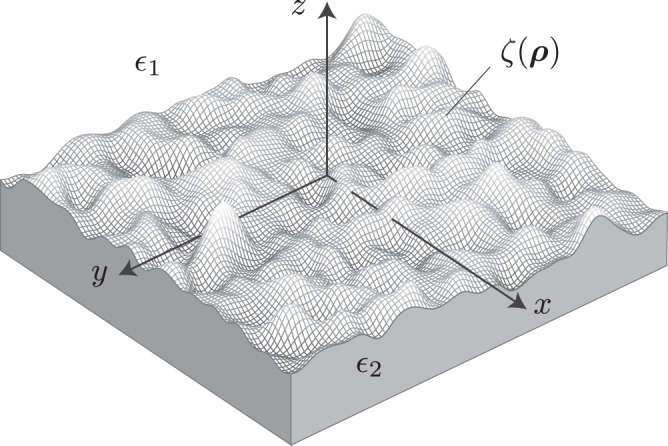
Schematic of a rough surface *ζ*(***ρ***) separating a dielectric and metal electric of permittivity *ε*_1_ and *ε*_2_ respectively.

We consider the system shown in Fig. 1 which is composed of a dielectric medium of electric permittivity *ε*_1_ in the region *z* > *ζ*(***ρ***) and a metal with complex permittivity *ε*_2_ in the region *z* < *ζ*(***ρ***), where ***ρ*** = (*x*, *y*, 0) denotes the in-plane position vector. Restriction is made to non-magnetic media for simplicity. The surface profile, *ζ*(***ρ***), is assumed to be an isotropic stationary stochastic process with zero mean 〈*ζ*(***ρ***)〉 = 0 and correlation function 〈*ζ*(***ρ***_1_)*ζ*(***ρ***_2_)〉 = *C*(|***ρ***_1_ − ***ρ***_2_|), where 〈…〉 denotes the average over the ensemble of surface profiles. This assumption aligns well with experimentally measured surface correlation functions^17^.

The electric field for a given realisation of *ζ* satisfies the wave equation ∇ × ∇ × **E**(**r**) − *ω*^2^*ε*(**r**)*μ*_0_**E**(**r**) = **0**, where *ε*(**r**) = *ε*_1_ + (*ε*_2_ − *ε*_1_)*H*(*ζ*(***ρ***) − *z*) is the electric permittivity distribution describing the rough surface, *H*(*z*) is the Heaviside function and an *e*^−*iωt*^ time dependence has been assumed. Letting *ε*(**r**) = *ε*_*f*_(**r**) + *δε*(**r**), the wave equation can be rewritten in the form ∇ × ∇ × **E**(**r**) − *ω*^2^*ε*_*f*_(**r**)*μ*_0_**E**(**r**) = *ω*^2^*μ*_0_*δε*(**r**)**E**(**r**). Here *ε*_*f*_(**r**) = *ε*_1_ + (*ε*_2_ − *ε*_1_)*H*(−*z*) describes the permittivity distribution for a flat interface at *z* = 0, which upon restricting to the small roughness regime simplifies to $\delta \varepsilon ({\bf{r}})\approx ({\varepsilon }_{2}-{\varepsilon }_{1})\delta (z)\zeta ({\boldsymbol{\rho }})+{\mathscr{O}}({\zeta }^{2})$. Formally the electric field distribution **E**(**r**) for a given realisation of surface roughness can then be found using the flat interface Green’s tensor ${\mathbb{G}}({\bf{r}},{\bf{r}}^{\prime} )$ according to

1 $${\bf{E}}({\bf{r}})={{\bf{E}}}_{0}({\bf{r}})+i\omega {\mu }_{0}\int {\mathbb{G}}({\bf{r}},{\bf{r}}^{\prime} ){\bf{J}}({\bf{r}}^{\prime} )d{\bf{r}}^{\prime} $$

where **E**_0_(**r**) is any solution to the homogeneous wave equation for a flat surface and the effect of surface roughness is accounted for by the equivalent current **J**(**r**) = −*iωδε*(**r**)**E**(**r**)^40,41^. Physically, **E**_0_ could for example represent an unscattered SPP propagating on a flat surface or an incident (and reflected) optical beam.

Our interest in this work lies in determining the two-point correlations present in roughness induced field fluctuations *δ***E**(**r**) = **E**(**r**) − 〈**E**(**r**)〉 as described by the matrix

2 $${\mathbb{W}}({{\bf{r}}}_{1},{{\bf{r}}}_{2})=\langle \delta {{\bf{E}}}^{\ast }({{\bf{r}}}_{1})\delta {{\bf{E}}}^{T}({{\bf{r}}}_{2})\rangle \mathrm{.}$$

Within the first order Born approximation (hence neglecting any multiple scattering) we note that the mean field distribution 〈**E**(**r**)〉 is simply **E**_0_(**r**), such that the correlation matrix can be found using Eqs (1 and 2) and is given by

3 $${\mathbb{W}}({{\bf{r}}}_{1},{{\bf{r}}}_{2})={\omega }^{2}{\mu }_{0}^{2}\iint {{\mathbb{G}}}^{\ast }({{\bf{r}}}_{1},{{\bf{r}}}_{3}){{\mathbb{W}}}_{J}({{\bf{r}}}_{3},{{\bf{r}}}_{4}){{\mathbb{G}}}^{T}({{\bf{r}}}_{2},{{\bf{r}}}_{4})d{{\bf{r}}}_{3}d{{\bf{r}}}_{4}$$

where ${{\mathbb{W}}}_{J}({{\bf{r}}}_{1},{{\bf{r}}}_{2})=\langle {{\bf{J}}}^{\ast }({{\bf{r}}}_{{\bf{1}}}){{\bf{J}}}^{{\bf{T}}}({{\bf{r}}}_{{\bf{2}}})\rangle$ is the two point correlation matrix for the equivalent surface current **J**(**r**) = −*iωδε*(**r**)**E**_0_(**r**). Since **E**_0_ is independent of the surface roughness profile by construction, the current correlation matrix can be expressed in the form

4 $${{\mathbb{W}}}_{J}({{\bf{r}}}_{1},{{\bf{r}}}_{2})={\omega }^{2}|{\varepsilon }_{2}-{\varepsilon }_{1}{|}^{2}\delta ({z}_{1})\delta ({z}_{2})C(P){{\bf{E}}}_{0}^{\ast }({{\bf{r}}}_{1}){{\bf{E}}}_{0}^{T}({{\bf{r}}}_{2})$$

where **P** = ***ρ***_1_ − ***ρ***_2_ = *P*[cos*θ*, sin*θ*, 0]^*T*^. Hitherto we have only accounted for randomness arising from the surface roughness, however, in reality the reference field **E**_0_ (assumed thus far to be time harmonic), may exhibit stochastic fluctuations in time, space or polarisation due to the nature of the exciting source. Assuming these fluctuations to be statistically independent of *ζ* and restricting to planar Schell-model sources^30^, we have ${{\mathbb{W}}}_{J}({{\bf{r}}}_{1},{{\bf{r}}}_{2})={{\mathbb{W}}}_{J}({\bf{P}})\delta ({z}_{1})\delta ({z}_{2})$ where

5 $${{\mathbb{W}}}_{J}({\bf{P}})={\omega }^{2}|{\varepsilon }_{2}-{\varepsilon }_{1}{|}^{2}C(P){{\mathbb{W}}}_{0}({\bf{P}}),$$

${{\mathbb{W}}}_{0}({\bf{P}})=\langle {{\bf{E}}}_{0}^{\ast }({\rho }_{1}){{\bf{E}}}_{0}^{T}({\rho }_{2})\rangle$ is the cross-spectral density matrix for the reference system and the ensemble average denoted by 〈…〉 now also includes averaging over the ensemble of sources.

To simplify Eq. (3) we note that the Green’s tensor ${\mathbb{G}}({{\bf{r}}}_{1},{{\bf{r}}}_{2})$ is only a function of the relative in-plane position vector ***ρ***_1_ − ***ρ***_2_ and can thus be expressed in the spatial frequency domain by means of a two-dimensional Fourier transform. With a little manipulation (see Supplementary Material) it follows that

6 $${\mathbb{W}}({\bf{P}},{z}_{1},{z}_{2})=\frac{{\omega }^{2}{\mu }_{0}^{2}}{{(2\pi )}^{2}}\iint d{\boldsymbol{\kappa }}\,d{\bf{Q}}\,{e}^{-i{\boldsymbol{\kappa }}\cdot ({\bf{P}}-{\bf{Q}})}{\mathop{{\mathbb{G}}}\limits^{ \sim }}^{\ast }({\boldsymbol{\kappa }},{z}_{1},0){{\mathbb{W}}}_{J}({\bf{Q}}){\mathop{{\mathbb{G}}}\limits^{ \sim }}^{T}({\boldsymbol{\kappa }},{z}_{2},0)$$

where we observe that ${\mathbb{W}}$ is a function of (**P**, *z*_1_, *z*_2_) only, **Q** = ***ρ***_3_ − ***ρ***_4_ = *Q*[cos*β*, sin*β*, 0]^*T*^ and ***κ*** = *κ*[cos*ϕ*, sin*ϕ*, 0]^*T*^. The Fourier domain Green’s function, $\mathop{{\mathbb{G}}}\limits^{ \sim }({\boldsymbol{\kappa }},{z}_{1},{z}_{2})$, for SPP scattering is given by^39^

7 $$\mathop{{\mathbb{G}}}\limits^{ \sim }({\boldsymbol{\kappa }},{z}_{1},{z}_{2})=\frac{4{\pi }^{2}\kappa {e}^{-\alpha \kappa ({z}_{1}+{z}_{2})}}{{K}_{0}({\gamma }^{2}-{\kappa }^{2})}[\hat{{\bf{z}}}-i\alpha \hat{{\boldsymbol{\kappa }}}][\hat{{\bf{z}}}+i\alpha \hat{{\boldsymbol{\kappa }}}{]}^{T}$$

where ${K}_{0}=2{\pi }^{2}\sqrt{-{\varepsilon }_{1}{\varepsilon }_{2}}(1-{\varepsilon }_{1}^{2}/{\varepsilon }_{2}^{2})({\varepsilon }_{1}+{\varepsilon }_{2})/({\varepsilon }_{1}{\varepsilon }_{2})$, $\alpha =\sqrt{-{\varepsilon }_{1}/{\varepsilon }_{2}}$, $\gamma ={k}_{0}\sqrt{{\varepsilon }_{1}{\varepsilon }_{2}/({\varepsilon }_{1}+{\varepsilon }_{2})}$ is the SPP propagation constant, *k*_0_ = *ω*/*c*, *c* is the speed of light in vacuum and hat notation is used to denote unit vectors e.g. $\hat{{\boldsymbol{\kappa }}}=\kappa /\kappa$. Expressing the correlation matrix ${{\mathbb{W}}}_{J}$ in the dyadic form

8 $${{\mathbb{W}}}_{J}({\bf{Q}})=\sum _{i=1}^{3}\sum _{j=1}^{3}{w}_{ij}({\bf{Q}}){\hat{{\bf{e}}}}_{i}(\beta ){\hat{{\bf{e}}}}_{j}^{T}(\beta )$$

where ${\hat{{\bf{e}}}}_{1}(\beta )=\hat{{\bf{Q}}}$, ${\hat{{\bf{e}}}}_{2}(\beta )=\hat{{\boldsymbol{\beta }}}$ and ${\hat{{\bf{e}}}}_{3}(\beta )=\hat{{\bf{z}}}$, and using Eq. (7), the integration in Eq. (6) can then be performed using standard integral identities^47^. For isotropic ${{\mathbb{W}}}_{0}$ we ultimately find

9 $${\mathbb{W}}({\bf{P}},{z}_{1},{z}_{2})=\frac{{(2\pi )}^{4}{\omega }^{2}{\mu }_{0}^{2}}{|{K}_{0}{|}^{2}}{\int }_{0}^{\infty }Q\{|\alpha {|}^{2}{w}_{11}(Q)+{w}_{33}(Q)\}{{\mathbb{V}}}_{0}(P,Q,\theta ,{z}_{1},{z}_{2})dQ$$

where (dropping the functional dependency for clarity)

10 $${{\mathbb{V}}}_{0}=\frac{1}{2}[\begin{array}{ccc}|\alpha {|}^{2}{L}_{-} & -|\alpha {|}^{2}{L}_{02}\,\sin \,2\theta  & 2{\alpha }^{\ast }{L}_{01}\,\cos \,\theta \\ -|\alpha {|}^{2}{L}_{02}\,\sin \,2\theta  & |\alpha {|}^{2}{L}_{+} & 2{\alpha }^{\ast }{L}_{01}\,\sin \,\theta \\ -2\alpha {L}_{01}\,\cos \,\theta  & -2\alpha {L}_{01}\,\sin \,\theta  & 2{L}_{00}\end{array}]$$

and *L*_±_ = *L*_00_ ± *L*_02_cos2*θ*. The *L*_*nm*_(*P*, *Q*, *z*_1_, *z*_2_) terms denote integrals over *κ* as detailed in the Supplementary Material. Closed form expressions for *L*_*nm*_ can however be found using Jordan’s lemma and Cauchy’s residue theorem^48^, yielding *L*_*nm*_ = *l*_*nm*_(*γ*) + *l*_*nm*_(−γ^*^), where

11 $${l}_{nm}(\kappa )=\frac{\pi }{4}\frac{{\sigma }^{n+m}{\kappa }^{2}{e}^{-\sigma \kappa {\mathscr{Z}}}}{\text{Im}[{\kappa }^{2}]}\{\begin{array}{cc}{J}_{n}(\kappa Q){H}_{m}^{(1)}(\kappa P) & {\rm{f}}{\rm{o}}{\rm{r}}\,P > Q\\ {H}_{n}^{(1)}(\kappa Q){J}_{m}(\kappa P) & {\rm{f}}{\rm{o}}{\rm{r}}\,P < Q\end{array}$$

${\mathscr{Z}}={\alpha }^{\ast }{z}_{1}+\alpha {z}_{2}$ and *σ*(*κ*) = sign[Re(*κ*)].

Equations (9–11) represent the central analytic result of this article. It is important to note, however, that the form of admissible ${{\mathbb{W}}}_{0}$ is limited through the assumption of in-plane isotropy, i.e. rotational invariance. Specifically ${{\mathbb{W}}}_{0}({\bf{P}})$ must have the form

12 $${{\mathbb{W}}}_{0}({\bf{P}})=[\begin{array}{ccc}{w}_{11}({\bf{P}}) & {w}_{12}({\bf{P}}) & 0\\ -{w}_{12}({\bf{P}}) & {w}_{11}({\bf{P}}) & 0\\ 0 & 0 & {w}_{33}({\bf{P}})\end{array}]$$

of which normally incident circularly polarised and unpolarised light are important examples. Although an angular dependence can be introduced into the form of ${{\mathbb{W}}}_{J}$ so as to allow a wider class of background fields to be described (ultimately giving rise to the presence of higher order Bessel functions in Eq. (9)), we have here elected to consider the most symmetric case so as not to overly complicate the mathematics. As evident from Eq. (9), the precise nature of correlations in the roughness induced field fluctuations is seen to be dependent on the source and surface correlation functions through *w*_11_ and *w*_33_, however they are insensitive to the off-diagonal elements *w*_12_.

## Results and Discussion

With Eqs (9–11) in hand, the remainder of this article focuses on studying some general and limiting properties of ${\mathbb{W}}$ in more detail. Our analysis begins with consideration of the polarisation properties of the plasmon speckle pattern. Since we consider an SPP field which both evanescently decays with distance from the surface and contains non-planar wavefronts due to random scattering, it is necessary to adopt a full three-dimensional (3D) formalism^49,50^ when discussing polarisation properties. In particular we consider the 3D spectral degree of cross polarisation (DOCP), ${{\mathscr{D}}}_{3\text{D}}({\bf{P}},{z}_{1},{z}_{2})$, defined by^49,51^

13 $${{\mathscr{D}}}_{n{\rm{D}}}^{2}({\bf{P}},{z}_{1},{z}_{2})=(3-\frac{n}{2})[\frac{\Vert {\mathbb{W}}({\bf{P}},{z}_{1},{z}_{2}){\Vert }_{F}^{2}}{{\rm{t}}{\rm{r}}{[{\mathbb{W}}({\bf{P}},{z}_{1},{z}_{2})]}^{2}}-\frac{1}{n}]$$

where ${\Vert \cdots \Vert }_{F}$ denotes the Frobenius matrix norm. Equation (13) also defines the two-dimensional (2D) DOCP (*n* = 2) as will be discussed below.

Initially restricting attention to **P** = **0**, whereby the DOCP is equivalent to the spectral degree of polarisation (DOP), we note that ${L}_{nm}(P=\mathrm{0,}\,Q,{z}_{1},{z}_{2})\sim {\delta }_{m0}$. It therefore follows that ${{\mathbb{V}}}_{0}\mathrm{(0,}\,Q,\theta ,{z}_{1},{z}_{2})={L}_{00}{\mathbb{A}}$ and ${\mathbb{W}}({\bf{0}},{z}_{1},{z}_{2})\sim {\mathbb{A}}$ where ${\mathbb{A}}={\rm{diag}}[|\alpha {|}^{2},|\alpha {|}^{2},\,2]$ is a constant diagonal matrix. Accordingly the DOCP is

14 $${{\mathscr{D}}}_{3{\rm{D}}}({\bf{0}},{z}_{1},{z}_{2})=\frac{|\alpha {|}^{2}-2}{2(1+|\alpha {|}^{2})}$$

which is independent of the axial position. We can similarly consider the 2D DOCP at **P** = **0** defined with respect to correlations between different in-plane field components (*E*_*x*_ and *E*_*y*_). By virtue of the assumed rotational invariance within the *x*-*y* plane, this is equivalent to correlations between the radial and azimuthal field components *E*_*ρ*_ and *E*_*θ*_). Moreover we can consider correlations between the axial and in-plane components. The associated 2 × 2 correlation matrices ${{\mathbb{W}}}_{\parallel }$ and ${{\mathbb{W}}}_{\perp }$ can be found by extracting the correct block matrices from ${\mathbb{W}}$. We find that ${{\mathscr{D}}}_{2{\rm{D}}}^{\parallel }=0$, showing that the in-plane field is unpolarised as would be expected from the assumed symmetry. Since SPPs on a flat surface are TM modes there is however always a non-zero out of plane field component in turn yielding a non-zero 2D DOCP, ${{\mathscr{D}}}_{2{\rm{D}}}^{\perp }$, satisfying $3{[{{\mathscr{D}}}_{2{\rm{D}}}^{\perp }({\bf{0}})]}^{2}=4{[{{\mathscr{D}}}_{3{\rm{D}}}({\bf{0}})]}^{2}-1$.

**Figure 2:**
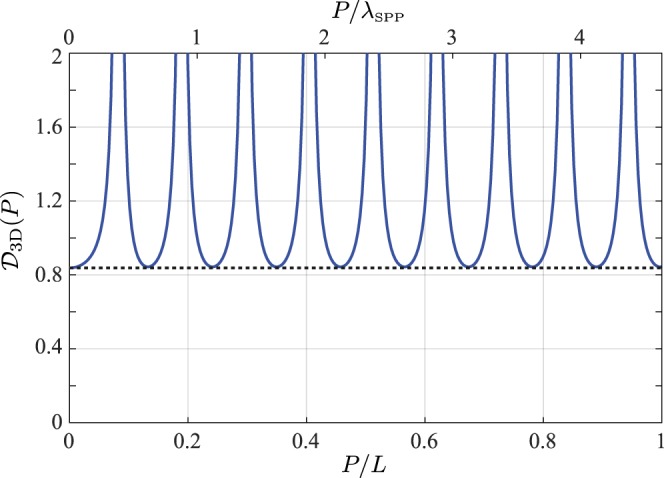
Degree of cross-polarisation, ${{\mathscr{D}}}_{3{\rm{D}}}$, for a SPP speckle field supported on a rough surface with Gaussian surface correlation function of correlation length *Q*_0_ = *λ*_SPP_/4. Separation coordinate has been normalised to both the SPP attenuation length *L* = 1/2Im[*γ*] (bottom axis) and SPP wavelength (top axis). Horizontal dashed line corresponds to limiting value given by Eq. (14).

An example of the behaviour of the spectral DOCP for two separated co-planar points (*P* > 0, *z*_1_ = *z*_2_ = 0) is shown in Fig. 2 assuming a rough gold-air interface (*ε*_1_ = 1, *ε*_2_ = −7.99 + 2.06*i* at a free-space wavelength of 600 nm^52^), normally incident circularly polarised light and a Gaussian surface correlation function $C(Q)={h}^{2}\exp [-{Q}^{2}/(2{Q}_{0}^{2})]$ of width *Q*_0_ = 0.25*λ*_SPP_, where *h*^2^ is the mean square height deviation of the surface and *λ*_SPP_ = 2*π*/Re[*γ*] is the SPP wavelength. We note that the DOCP is independent of *h* since it appears as a common factor in the numerator and denominator in Eq. (13) and hence cancels. It is evident from Fig. 2 that the zero-separation DOCP (given by Eq. (14) and shown by the dashed line) acts as a lower bound on ${{\mathscr{D}}}_{3{\rm{D}}}$ for all separations *P* ≥ 0. Moreover the DOCP regularly takes this limiting value with a period given by the SPP propagation wavelength. We also note that since ${\mathbb{W}}({\bf{P}},{z}_{1},{z}_{2})$ is not non-negative definite for **P** ≠ **0**, the 3D DOCP can adopt values greater than unity in contrast to the spectral DOP ${{\mathscr{D}}}_{3{\rm{D}}}({\bf{0}})$^51^. Infinite values are indeed seen in Fig. 2 corresponding to separations for which the points have zero degree of coherence (see below), i.e., where the denominator in Eq. (13) is zero.

A further common metric parametrising random fields is the so-called spectral degree of coherence (DOC), which provides a measure of fringe visibility in interferometric measurements and is thus pertinent to the growing number of plasmon interferometers^42,43^. The DOC is defined here with respect to the correlation matrix at ***ρ***_1_ = ***ρ***_2_ = **0** and we restrict to the *z*_1_ = *z*_2_ = *z*_0_ plane such that $\mu ({\bf{P}},{z}_{0})={\rm{t}}{\rm{r}}[{\mathbb{W}}({\bf{P}},{z}_{0},{z}_{0})]/{\rm{t}}{\rm{r}}[{\mathbb{W}}({\bf{0}},\,0,\,0)]$^53^. To evaluate the DOC we first note that ${\rm{tr}}[{{\mathbb{V}}}_{0}]=(|\alpha {|}^{2}+1){L}_{00}$ and partition the integration domain in Eq. (7) into the two regions defined by 0 ≤ *Q* < *P* and *P* ≤ *Q* ≤ ∞. The general form of the DOC, given in the Supplementary Material, is dictated by the functional form of the surface and source correlation functions through *w*_11_ and *w*_33_. In contrast to the universal, i.e. surface and source independent, characteristics of speckle patterns in the far field^22^, non-universal correlations are thus exhibited in random SPP scattering. Similar non-universal behaviour in near field speckle patterns has been previously discussed^19^, and may be useful for imaging or sensing^5,9^. Noting, however, that typically ${\rm{Im}}[\gamma ]\ll {\rm{Re}}[\gamma ]$, that is to say the SPP propagation length (*L* = 1/2Im[*γ*]) is much larger than the wavelength, we can approximate the DOC according to

15 $$\,\,\,\mu ({\bf{P}},{z}_{0})\approx \frac{{e}^{-\gamma {{\mathscr{Z}}}_{0}}}{2}\{[{H}_{0}^{(1)}(\gamma P)-{H}_{0}^{(1)}(-{\gamma }^{\ast }P)]{f}_{J}^{P}+{J}_{0}(\gamma P)[{f}_{J}^{{\rm{\infty }}}+i{f}_{Y}^{{\rm{\infty }}}]+{J}_{0}(-{\gamma }^{\ast }P)[{f}_{J}^{{\rm{\infty }}}-i{f}_{Y}^{{\rm{\infty }}}]\},$$

where the *f* factors describe the ratio of integrals defined in the Supplementary Material and ${{\mathscr{Z}}}_{0}=2\,{\rm{Re}}[\alpha ]{z}_{0}$. For our purposes here, the precise form of the integrals is unimportant except to note that the integrand is dependent on *w*_11_ and *w*_33_.

A number of conclusions can be made on the basis of Eq. (15). Firstly, for a loss-free metal *ε*_2_ is purely real and negative and consequently the SPP propagation constant *γ* is real. Using the reflection formulae for the Bessel and Hankel functions^54^ it follows that the DOC reduces to the universal form *μ*(**P**) = *J*_0_(*γP*) which decays as $\sim {P}^{-\mathrm{1/2}}$. The Bessel function dependence of the DOC is familiar from the case of 2D scalar waves, however, we note we have derived it here using the full electromagnetic expressions for SPP waves. Physically, however, all metals exhibit some loss and it is not reasonable to neglect absorption. Instead we observe that typical surface and/or source correlation functions decay over some characteristic correlation length *Q*_0_. When considering the DOC between points separated by distances greater than *Q*_0_ length, by virtue of the dependence of the *f* terms on *w*_11_ and *w*_33_ it can be shown that ${f}_{J}^{\infty }\approx {f}_{Y}^{\infty }\approx 0$ and ${f}_{J}^{P}\approx 1$, such that

16 $$\mu ({\bf{P}},{z}_{0})\approx \frac{{e}^{-\gamma {{\mathscr{Z}}}_{0}}}{2}[{H}_{0}^{\mathrm{(1)}}(\gamma P)-{H}_{0}^{\mathrm{(1)}}(\,-\,{\gamma }^{\ast }P)]\mathrm{.}$$

**Figure 3:**
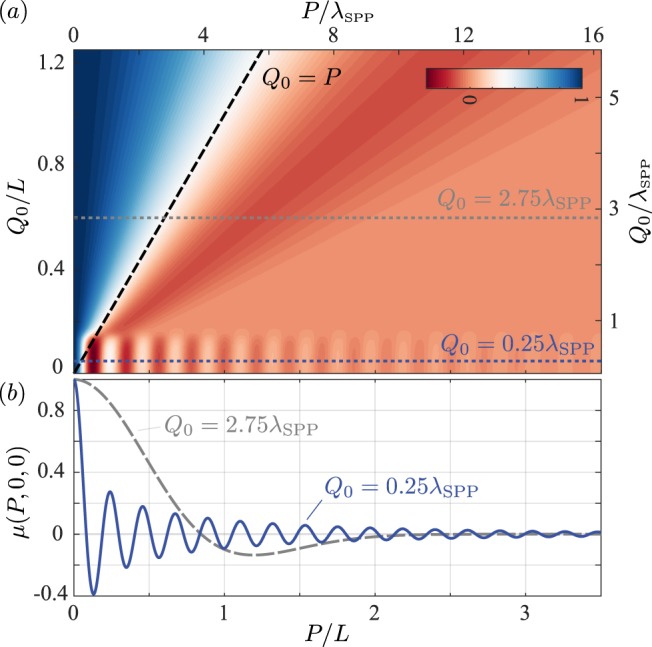
(**a**) Degree of coherence *μ*(*P*, 0, 0) as a function of surface correlation length *Q*_0_. Horizontal dashed lines correspond to cross-sections plotted in (**b**).

This analytic form is notably independent of the precise nature of the correlations ${{\mathbb{W}}}_{J}$. For separations $P\lesssim {Q}_{0}$, however, non-universal behaviour of *μ* is manifest. Nevertheless in the limit that *Q*_0_ → 0, corresponding to an infinitesimally short surface correlation length, or use of a thermal source^33^, the DOC is described by Eq. (16) for all *P* > 0. This behaviour can be seen in Fig. 3(a) where we have plotted the DOC calculated numerically from Eq. (9) as a function of both the spatial separation *P* and the surface correlation length *Q*_0_. All other simulation parameters are the same as in Fig. 2. Specifically we note that for correlation lengths ${{\rm{Q}}}_{0}\lesssim {\lambda }_{{\rm{SPP}}}\mathrm{/2}$ the functional behaviour described by Eq. (16) is apparent, whereas for correlation lengths larger than $\sim {\lambda }_{{\rm{SPP}}}$ the DOC is dictated by the correlation function ${{\mathbb{W}}}_{J}$. Whilst in this latter case Eq. (16) holds at large separations, the DOC has nevertheless fallen essentially to zero such that the SPP oscillations are not visible. Cross-sections of *μ*(*P*, 0, 0) for *Q*_0_ = 0.25*λ*_SPP_ and 2.75*λ*_SPP_ (as depicted by the horizontal dashed lines in Fig. 3(a)) are also shown in Fig. 3(b), which demonstrate this differing behaviour. Finally, we observe that there exists a lower limit to the scale of fluctuations in SPP fields as can be seen in Fig. 3 and is captured in Eq. (16). In particular, the minimum fluctuation length is on the order of the surface plasmon wavelength. This behaviour is notably different to that of near field speckle patterns comprised of evanescent waves for which sub-wavelength correlation lengths can occur^15^. The difference in behaviour can be simply understood by noting that in near field speckle, a range of waves with differing transverse wavenumbers *κ* contribute to the total field, whereas in SPP speckle the resonant nature of SPPs means only a single transverse wavenumber is present and thus the the frequency of fluctuations in lower bounded.

## Conclusions

In summary, this work has considered field correlations present in surface plasmon speckle patterns arising from scattering of SPPs propagating on a rough surface. Within the first order Born approximation and assuming in-plane statistical isotropy and homogeneity, we have derived closed form analytic expressions for the correlation matrix for roughness induced field fluctuations relative to a smooth metallo-dielectric interface, as given by Eqs (9–11). Correlations were thereby found to be independent of off-diagonal elements of the source spectral density matrix and to exponentially decay with distance from the interface. Analytic formulae derived also allowed us to investigate the polarisation and coherence properties of SPP speckle patterns in closer detail. In particular, we found that the 3D DOP is not only independent of the axial distance from the interface, but that it also serves as a lower bound for the DOCP across the whole speckle distribution. Universal forms for the DOC were also shown to arise for low loss metals or when the correlation length of the surface height, or source field were small (<*λ*_SPP_/2). Non-universal behaviour can however result when such conditions are not met whereby the DOC (and also the correlation matrix ${\mathbb{W}}$) is dominated by the source/surface correlation function. A minimum fluctuation length, on the order of the SPP wavelength, was thus found in contrast to purely evanescent near field speckle patterns.

## Supplementary information

Supplementary Derivations
