# Supplementary material for: Field Correlations in Surface Plasmon Speckle
Source: Sci Rep. 2019 Jun 7;9:8359. doi: 10.1038/s41598-019-44780-5 (PMC6555795; doi:10.1038/s41598-019-44780-5)
Supplement: Supplementary file 1 — Supplementary Derivations [file 41598_2019_44780_MOESM1_ESM.pdf]

Field Correlations in Surface Plasmon Speckle - Supplementary Material

Matthew R. Foreman[1,*]

[1]Blackett Laboratory, Imperial College London, Prince Consort Road, London, SW7 2AZ, United Kingdom
*matthew.foreman@imperial.ac.uk

S1 Derivation of field correlation matrix

Within the electrostatic approximation the electric field of a surface plasmon polariton (SPP) propagating on a surface defined by the surface profile $\zeta(\rho)$, where ρ denotes a position vector in the x-y plane, is given by $\mathbf{E}(\mathbf{r}) = -\nabla\phi(\mathbf{r})$. The scalar potential ϕ is a solution to Laplace's equation $\nabla^2\phi = 0$ subject to continuity constraints on ϕ and its normal derivative on the supporting interface $\zeta(\rho)$[1]. The general form of the associated vector Helmholtz equation for the SPP field $\mathbf{E}$ in a source free region can be written in the form

$$\mu(\mathbf{r})\nabla\times\frac{1}{\mu(\mathbf{r})}\nabla\times\mathbf{E}(\mathbf{r}) - \omega^2\varepsilon(\mathbf{r})\mu(\mathbf{r})\mathbf{E}(\mathbf{r}) = \mathbf{0}, \tag{S1}$$

where $\mu(\mathbf{r})$ and $\varepsilon(\mathbf{r})$ are the spatial distributions of the magnetic permeability and electric permittivity respectively and ω is the angular frequency of the wave (assumed monochromatic with an $\exp[-i\omega t]$ time dependence). We henceforth shall consider only non-magnetic media for which $\mu(\mathbf{r}) = \mu_0$ for all $\mathbf{r}$. The electric permittivity distribution describing an arbitrary surface can be written in the form

$$\varepsilon(\mathbf{r}) = \varepsilon_1 + (\varepsilon_2 - \varepsilon_1)H(\zeta(\rho) - z) \tag{S2}$$

where $H(z)$ is the Heaviside function. We also define the permittivity difference $\delta\varepsilon(\mathbf{r}) = \varepsilon(\mathbf{r}) - \varepsilon_f(\mathbf{r})$ relative to the permittivity distribution for a flat surface

$$\varepsilon_f(\mathbf{r}) = \varepsilon_1 + (\varepsilon_2 - \varepsilon_1)H(-z). \tag{S3}$$

Rearranging Eq. (S1) thus gives

$$\nabla\times\nabla\times\mathbf{E}(\mathbf{r}) - \omega^2\varepsilon_f(\mathbf{r})\mu_0\mathbf{E}(\mathbf{r}) = \omega^2\mu_0\delta\varepsilon(\mathbf{r})\mathbf{E}(\mathbf{r}). \tag{S4}$$

The roughness can thus be modelled as an equivalent source current $\mathbf{J}(\mathbf{r}) = -i\omega\delta\varepsilon(\mathbf{r})\mathbf{E}(\mathbf{r})$. The general solution for the scattered SPP field can be written directly by employing the Green's tensor $\mathbb{G}(\mathbf{r}_1,\mathbf{r}_2)$ describing elastic scattering of SPPs on a flat surface (defined below).

If the underlying surface profile is a random field, then so too is the electric field $\mathbf{E}$. Random fields can be partially characterised by their second order moments and we thus consider the two-point field correlation matrix $\mathbb{W}(\mathbf{r}_1,\mathbf{r}_2) = \langle\delta\mathbf{E}^*(\mathbf{r}_1)\delta\mathbf{E}^T(\mathbf{r}_2)\rangle$ here, where $\delta\mathbf{E}(\mathbf{r}) = \mathbf{E}(\mathbf{r}) - \langle\mathbf{E}(\mathbf{r})\rangle$. Upon averaging Eq. (S4) we find the mean field $\langle\mathbf{E}(\mathbf{r})\rangle$ satisfies the equation

$$\nabla\times\nabla\times\langle\mathbf{E}(\mathbf{r})\rangle - \omega^2\varepsilon_f(\mathbf{r})\mu_0\langle\mathbf{E}(\mathbf{r})\rangle = \omega^2\mu_0\langle\delta\varepsilon(\mathbf{r})\mathbf{E}(\mathbf{r})\rangle. \tag{S5}$$

Making a Born approximation, the driving field appearing in $\mathbf{J}$ is replaced by the incident field $\mathbf{E}_0$ which is the solution of Eq. (S1) with $\varepsilon \to \varepsilon_f$, such that $\langle\delta\varepsilon(\mathbf{r})\mathbf{E}(\mathbf{r})\rangle \to \langle\delta\varepsilon(\mathbf{r})\mathbf{E}_0(\mathbf{r})\rangle = \langle\delta\varepsilon(\mathbf{r})\rangle\langle\mathbf{E}_0(\mathbf{r})\rangle = \mathbf{0}$ since $\langle\delta\varepsilon(\mathbf{r})\rangle = 0$ by definition. Hence subtracting Eqs. (S4) and (S5) we have

$$\nabla\times\nabla\times\delta\mathbf{E}(\mathbf{r}) - \omega^2\varepsilon_f(\mathbf{r})\mu_0\delta\mathbf{E}(\mathbf{r}) = \omega^2\mu_0\delta\varepsilon(\mathbf{r})\mathbf{E}_0(\mathbf{r}) \tag{S6}$$

which has the solution

$$\delta\mathbf{E}(\mathbf{r}_1) = i\omega\mu_0\int\mathbb{G}(\mathbf{r}_1,\mathbf{r}_2)\mathbf{J}(\mathbf{r}_2)d\mathbf{r}_2, \tag{S7}$$

where we now introduce subscripts on the position vectors to denote different variables. Using Eq. (S7) it follows that

$$\mathbb{W}(\mathbf{r}_1,\mathbf{r}_2) = \omega^2\mu_0^2\iint\mathbb{G}^*(\mathbf{r}_1,\mathbf{r}_3)\mathbb{W}_J(\mathbf{r}_3,\mathbf{r}_4)\mathbb{G}^T(\mathbf{r}_2,\mathbf{r}_4)d\mathbf{r}_3d\mathbf{r}_4 \tag{S8}$$

where $\mathbb{W}_J(\mathbf{r}_1,\mathbf{r}_2) = \langle \mathbf{J}^*(\mathbf{r}_1)\mathbf{J}^T(\mathbf{r}_2)\rangle$ is the two point correlation matrix for the current $\mathbf{J}(\mathbf{r}) = -i\omega\delta\varepsilon(\mathbf{r})\mathbf{E}_0(\mathbf{r})$.

In the small roughness regime (i.e $\langle \zeta(\mathbf{0})^2\rangle \ll \lambda^2$) the permittivity difference can be expanded viz. $\delta\varepsilon(\mathbf{r}) \approx (\varepsilon_2 - \varepsilon_1)\delta(z)\zeta(\rho) + \mathscr{O}(\zeta^2)$, whereby we can write $\mathbf{J}(\mathbf{r}) = \mathbf{j}(\rho)\delta(z)$. We assume that $\mathbf{j}(\rho)$ is statistically homogeneous within the plane. With these assumptions we can write

$$\mathbb{W}_J(\mathbf{r}_1,\mathbf{r}_2) = \langle \mathbf{j}(\rho_1)\mathbf{j}^T(\rho_2)\rangle\delta(z_1)\delta(z_2) = \mathbb{w}_J(\rho_1,\rho_2)\delta(z_1)\delta(z_2). \tag{S9}$$

Statistical transverse homogeneity of $\mathbf{j}(\rho)$ implies that $\mathbb{W}_J(\mathbf{r}_1,\mathbf{r}_1+\mathbf{P}) = \mathbb{w}_J(\mathbf{P})\delta(z_1)\delta(z_2)$ where $\mathbf{P}$ is an arbitrary vector lying in the x-y plane. Switching to a Fourier representation will allow simplification of the integrals required to evaluate $\mathbb{W}(\mathbf{r}_1,\mathbf{r}_2)$. Given the symmetry assumed in our system we will consider two-dimensional Fourier transforms. We thus define

$$\widetilde{\mathbf{E}}(\kappa,z) = \int \mathbf{E}(\rho,z)e^{-i\kappa\cdot\rho}d\rho \tag{S10}$$

$$\widetilde{\mathbf{J}}(\kappa,z) = \delta(z)\widetilde{\mathbf{j}}(\kappa) = \delta(z)\int \mathbf{j}(\rho)e^{-i\kappa\cdot\rho}d\rho. \tag{S11}$$

Consider then

$$\widetilde{\mathbb{W}}_J(\kappa_1,\kappa_2,z_1,z_2) = \iint \mathbb{W}_J(\mathbf{r}_1,\mathbf{r}_2)e^{i\kappa_1\cdot\rho_1}e^{-i\kappa_2\cdot\rho_2}d\rho_1 d\rho_2 \tag{S12}$$

$$= \delta(z_1)\delta(z_2)\iint \mathbb{w}_J(\rho_1,\rho_2)e^{i\kappa_1\cdot\rho_1}e^{-i\kappa_2\cdot\rho_2}d\rho_1 d\rho_2 \tag{S13}$$

$$= \delta(z_1)\delta(z_2)\left\langle \int \mathbf{j}^*(\rho_1)e^{i\kappa_1\cdot\rho_1}d\rho_1 \int \mathbf{j}^T(\rho_2)e^{-i\kappa_2\cdot\rho_2}d\rho_2 \right\rangle \tag{S14}$$

$$= \delta(z_1)\delta(z_2)\left\langle \widetilde{\mathbf{j}}^*(\kappa_1)\widetilde{\mathbf{j}}^T(\kappa_2)\right\rangle \tag{S15}$$

$$= \delta(z_1)\delta(z_2)\widetilde{\mathbb{w}}_J(\kappa_1,\kappa_2). \tag{S16}$$

We know, however, that for a 2D statistically homogeneous random field that $\mathbb{w}_J(\rho_1,\rho_2) = \mathbb{w}_J(\rho_1-\rho_2)$. Consider then the form of

$$\widetilde{\mathbb{w}}_J(\kappa_1,\kappa_2) = \iint \mathbb{w}_J(\rho_1-\rho_2)e^{i\kappa_1\cdot\rho_1}e^{-i\kappa_2\cdot\rho_2}d\rho_1 d\rho_2. \tag{S17}$$

Letting $\mathbf{P} = \rho_1 - \rho_2$ we can perform a change of variables to give

$$\widetilde{\mathbb{w}}_J(\kappa_1,\kappa_2) = \iint \mathbb{w}_J(\mathbf{P})e^{i\kappa_1\cdot\rho_1}e^{-i\kappa_2\cdot[\rho_1-\mathbf{P}]}d\rho_1 d\mathbf{P} \tag{S18}$$

$$= \int e^{i[\kappa_1-\kappa_2]\cdot\rho_1}d\rho_1 \int \mathbb{w}_J(\mathbf{P})e^{+i\kappa_2\cdot\mathbf{P}}d\mathbf{P} \tag{S19}$$

$$= (2\pi)^2\delta(\kappa_1-\kappa_2)\widetilde{\mathbb{w}}_J(-\kappa_2). \tag{S20}$$

The SPP Green's tensor for a planar interface is a function of $\mathbf{P} = \rho_1 - \rho_2$ only[2,3]. We can therefore write the Green's tensor in the form

$$\mathbb{G}(\mathbf{r}_1,\mathbf{r}_2) = \mathbb{G}(\rho_1,\rho_2,z_1,z_2) = \mathbb{G}(\mathbf{P},z_1,z_2) = \frac{1}{(2\pi)^2}\int \widetilde{\mathbb{G}}(\kappa,z_1,z_2)e^{i\kappa\cdot\rho}d\kappa. \tag{S21}$$

The electric field two-point correlation matrix can hence be written using Eqs. (S8) and (S21) as

$$\mathbb{W}(\mathbf{r}_1,\mathbf{r}_2) = \frac{\omega^2\mu_0^2}{(2\pi)^4}\iint d\mathbf{r}_3 d\mathbf{r}_4 \left[\int \widetilde{\mathbb{G}}^*(\kappa_1,z_1,z_3)e^{-i\kappa_1\cdot[\rho_1-\rho_3]}d\kappa_1\right]\mathbb{W}_J(\mathbf{r}_3,\mathbf{r}_4)\left[\int \widetilde{\mathbb{G}}^T(\kappa_2,z_2,z_4)e^{i\kappa_2\cdot[\rho_2-\rho_4]}d\kappa_2\right]. \tag{S22}$$

Rearranging and reordering the integration gives

$$\mathbb{W}(\mathbf{r}_1,\mathbf{r}_2) = \frac{\omega^2\mu_0^2}{(2\pi)^4}\iint d\kappa_1 d\kappa_2 e^{-i\kappa_1\cdot\rho_1}e^{i\kappa_2\cdot\rho_2}\widetilde{\mathbb{G}}^*(\kappa_1,z_1,z_3)\left[\iint \mathbb{W}_J(\mathbf{r}_3,\mathbf{r}_4)e^{i\kappa_1\cdot\rho_3}e^{-i\kappa_2\cdot\rho_4}d\mathbf{r}_3 d\mathbf{r}_4\right]\widetilde{\mathbb{G}}^T(\kappa_2,z_2,z_4). \tag{S23}$$

Further using Eqs. (S12), (S16) and (S20) this expression reduces to

$$\mathbb{W}(\mathbf{r}_1,\mathbf{r}_2) = \frac{\omega^2\mu_0^2}{(2\pi)^2}\iint d\kappa_1 d\kappa_2 e^{-i\kappa_1\cdot\rho_1}e^{i\kappa_2\cdot\rho_2}\delta(\kappa_1-\kappa_2)\iint dz_3dz_4\widetilde{\mathbb{G}}^*(\kappa_1,z_1,z_3)\delta(z_3)\delta(z_4)\widetilde{\mathbb{w}}_J(-\kappa_1)\widetilde{\mathbb{G}}^T(\kappa_2,z_2,z_4). \tag{S24}$$

The Dirac delta functions allow the integration of z_3, z_4 and κ_2 to be easily performed, yielding

$$\mathbb{W}(\mathbf{r}_1,\mathbf{r}_2) = \frac{\omega^2\mu_0^2}{(2\pi)^2}\int d\kappa\, e^{-i\kappa\cdot\mathbf{P}}\widetilde{\mathbb{G}}^*(\kappa,z_1,0)\widetilde{\mathbb{w}}_J(-\kappa)\widetilde{\mathbb{G}}^T(\kappa,z_2,0) \tag{S25}$$

$$= \frac{\omega^2\mu_0^2}{(2\pi)^2}\iint d\kappa\, d\mathbf{Q}\, e^{-i\kappa\cdot(\mathbf{P}-\mathbf{Q})}\widetilde{\mathbb{G}}^*(\kappa,z_1,0)\mathbb{w}_J(\mathbf{Q})\widetilde{\mathbb{G}}^T(\kappa,z_2,0), \tag{S26}$$

where we have also dropped the unneeded subscript on κ_1 and used the definition of $\widetilde{\mathbb{w}}_J(-\kappa)$ in the final equality.

The SPP contribution to the total Green's tensor for a single planar interface is given by Eq. (21) of[2]. Specifically assuming an interface at $z=0$ separates a dielectric medium ($z>0$) with permittivity ε_1 and a metallic medium ($z<0$) of complex permittivity ε_2 ($\mathrm{Im}[\varepsilon_2]>0$) the SPP Green's tensor can be shown to take the form

$$\mathbb{G}(\mathbf{r}_1,\mathbf{r}_2) = \int_0^\infty \frac{\kappa^2}{K_0(k_{\mathrm{SPP}}^2-\kappa^2)}d\kappa\int_0^{2\pi}d\phi\left[\hat{\mathbf{z}}-i\alpha\hat{\kappa}\right]\left[\hat{\mathbf{z}}^T+i\alpha\hat{\kappa}^T\right]e^{i\kappa\cdot\mathbf{P}}e^{-\alpha\kappa(z_1+z_2)} \tag{S27}$$

for $z_1,z_2\geq 0$, where $\alpha=\sqrt{-\varepsilon_1/\varepsilon_2}$, $\kappa=\kappa[\cos\phi,\sin\phi,0]^T$, $\hat{\kappa}=\kappa/\kappa$,

$$K_0 = 2\pi^2\sqrt{-\varepsilon_1\varepsilon_2}\left(1-\frac{\varepsilon_1^2}{\varepsilon_2^2}\right)\frac{\varepsilon_1+\varepsilon_2}{\varepsilon_1\varepsilon_2}, \tag{S28}$$

$k_{\mathrm{SPP}}=k_0\sqrt{\varepsilon_1\varepsilon_2/(\varepsilon_1+\varepsilon_2)}$, $k_0=\omega/c$ and c is the speed of light in vacuum. Comparison with Eq. (S21) yields

$$\widetilde{\mathbb{G}}(\kappa,z_1,z_2) = \frac{(2\pi)^2\kappa}{K_0(k_{\mathrm{SPP}}^2-\kappa^2)}e^{-\alpha\kappa(z_1+z_2)}\left[\hat{\mathbf{z}}-i\alpha\hat{\kappa}\right]\left[\hat{\mathbf{z}}^T+i\alpha\hat{\kappa}^T\right] = g(\kappa,z_1,z_2)\mathbb{M}(\phi) \tag{S29}$$

where $g(\kappa,z_1,z_2)=(2\pi)^2\kappa e^{-\alpha\kappa(z_1+z_2)}/[K_0(k_{\mathrm{SPP}}^2-\kappa^2)]$ and

$$\mathbb{M}(\phi) = \left[\hat{\mathbf{z}}-i\alpha\hat{\kappa}\right]\left[\hat{\mathbf{z}}^T+i\alpha\hat{\kappa}^T\right] = \hat{\mathbf{z}}\hat{\mathbf{z}}^T+i\alpha[\hat{\mathbf{z}}\hat{\kappa}^T+\hat{\kappa}\hat{\mathbf{z}}^T]-\alpha^2\hat{\kappa}\hat{\kappa}^T$$

$$= \begin{bmatrix} \frac{1}{2}\alpha^2(1+\cos 2\phi) & \frac{1}{2}\alpha^2\sin 2\phi & -i\alpha\cos\phi \\ \frac{1}{2}\alpha^2\sin 2\phi & \frac{1}{2}\alpha^2(1-\cos 2\phi) & -i\alpha\sin\phi \\ i\alpha\cos\phi & i\alpha\sin\phi & 1 \end{bmatrix}. \tag{S30}$$

Expressing $\mathbb{w}_J(\mathbf{Q})$ in the form

$$\mathbb{w}_J(\mathbf{Q}) = \sum_{j=1}^{3}\sum_{k=1}^{3} w_{jk}(\mathbf{Q})\hat{\mathbf{e}}_j(\beta)\hat{\mathbf{e}}_k^T(\beta) \tag{S31}$$

where $\mathbf{Q}=Q[\cos\beta,\sin\beta,0]^T$ and

$$\hat{\mathbf{e}}_1(\beta) = \hat{\mathbf{Q}} = \cos\beta\hat{\mathbf{x}}+\sin\beta\hat{\mathbf{y}} \tag{S32}$$

$$\hat{\mathbf{e}}_2(\beta) = \hat{\beta} = -\sin\beta\hat{\mathbf{x}}+\cos\beta\hat{\mathbf{y}} \tag{S33}$$

$$\hat{\mathbf{e}}_3(\beta) = \hat{\mathbf{z}}, \tag{S34}$$

are unit vectors in the radial, azimuthal and longitudinal directions respectively, we can use Eq. (S29) to give

$$\mathbb{W}(\mathbf{r}_1,\mathbf{r}_2) = \frac{(2\pi)^2\omega^2\mu_0^2}{|K_0|^2}\sum_{j,k=1}^{3}\int w_{jk}(\mathbf{Q})\mathbb{I}_{jk}(\mathbf{P}-\mathbf{Q})d\mathbf{Q}, \tag{S35}$$

where letting $\mathscr{Z} = \alpha^* z_1 + \alpha z_2$

$$\mathbb{I}_{jk}(\mathbf{P}-\mathbf{Q}, z_1, z_2) = \int_0^\infty \int_0^{2\pi} \frac{\kappa^3 e^{-i\kappa\cdot(\mathbf{P}-\mathbf{Q})} e^{-\kappa \mathscr{Z}} d\kappa d\phi}{(k_{\mathrm{SPP}}^2 - \kappa^2)(k_{\mathrm{SPP}}^2 - \kappa^2)^*} \mathbb{M}^*(\phi)\hat{\mathbf{e}}_j(\beta)\hat{\mathbf{e}}_k^T(\beta)\mathbb{M}^T(\phi). \tag{S36}$$

The matrix factor can be evaluated and takes the form

$$\mathbb{M}_{jk}(\mathbf{P}-\mathbf{Q}) = \mathbb{M}^*(\phi)\hat{\mathbf{e}}_j(\beta)\hat{\mathbf{e}}_k^T(\beta)\mathbb{M}^T(\phi) = m_{jk}\mathscr{M}_{jk}(\beta,\phi)\mathbb{M}_{33}(\phi) \tag{S37}$$

where

$$\mathbb{M}_{33}(\phi) = \begin{bmatrix} \frac{1}{2}|\alpha|^2(1+\cos 2\phi) & \frac{1}{2}|\alpha|^2 \sin 2\phi & i\alpha^* \cos\phi \\ \frac{1}{2}|\alpha|^2 \sin 2\phi & \frac{1}{2}|\alpha|^2(1-\cos 2\phi) & i\alpha^* \sin\phi \\ -i\alpha\cos\phi & -i\alpha\sin\phi & 1 \end{bmatrix} \tag{S38}$$

$m_{11} = m_{22} = \frac{1}{2}|\alpha|^2$, $m_{12} = m_{21}^* = -\frac{1}{2}|\alpha|^2$, $m_{13} = m_{31}^* = -i\alpha^*$, $m_{23} = m_{32}^* = i\alpha^*$, $m_{33} = 1$ and

$$\mathscr{M}_{11}(\beta,\phi) = 1 + \cos 2(\beta-\phi) \tag{S39}$$
$$\mathscr{M}_{22}(\beta,\phi) = 1 - \cos 2(\beta-\phi) \tag{S40}$$
$$\mathscr{M}_{33}(\beta,\phi) = 1 \tag{S41}$$
$$\mathscr{M}_{12}(\beta,\phi) = \mathscr{M}_{21}(\beta,\phi) = \sin 2(\beta-\phi) \tag{S42}$$
$$\mathscr{M}_{13}(\beta,\phi) = \mathscr{M}_{31}(\beta,\phi) = \cos(\beta-\phi) \tag{S43}$$
$$\mathscr{M}_{23}(\beta,\phi) = \mathscr{M}_{32}(\beta,\phi) = \sin(\beta-\phi). \tag{S44}$$

At this point we make the further restriction that $w_{jk}(\mathbf{Q}) = w_{jk}(Q)$, i.e. the two-point current correlation matrix is a function of the separation of the two points only and not their relative direction. For this case we have

$$\mathbb{W}(\mathbf{r}_1,\mathbf{r}_2) = \frac{(2\pi)^2\omega^2\mu_0^2}{|K_0|^2} \sum_{j,k=1}^{3} m_{jk} \int_0^\infty Q w_{jk}(Q) \mathbb{I}_{jk}^{\beta}(\mathbf{P}-\mathbf{Q}, z_1, z_2) dQ, \tag{S45}$$

where using Eq. (S37), letting $\mathbf{P} = P[\cos\theta, \sin\theta, 0]^T$ and splitting the exponential term we can write

$$\mathbb{I}_{jk}^{\beta}(\mathbf{P}-\mathbf{Q}, z_1, z_2) = \int_0^\infty \frac{\kappa^3 e^{-\kappa\mathscr{Z}}}{(k_{\mathrm{SPP}}^2 - \kappa^2)(k_{\mathrm{SPP}}^2 - \kappa^2)^*} \int_0^{2\pi} e^{-i\kappa P\cos(\theta-\phi)} \mathbb{M}_{33}(\phi) \left[\int_0^{2\pi} \mathscr{M}_{jk}(\beta,\phi) e^{i\kappa Q\cos(\beta-\phi)} d\beta\right] d\phi d\kappa. \tag{S46}$$

Recalling the integration identity[4]

$$\int_0^{2\pi} \left\{ \begin{array}{c} \cos n\beta \\ \sin n\beta \end{array} \right\} e^{-ia\cos(\beta-\gamma)} d\beta = 2\pi(-i)^n \left\{ \begin{array}{c} \cos n\gamma \\ \sin n\gamma \end{array} \right\} J_n(a). \tag{S47}$$

the angular integration over β in Eq. (S46) can be performed yielding

$$\begin{aligned} 2\pi B_{jk} &= \int_0^{2\pi} \mathscr{M}_{jk}(\beta,\phi) e^{i\kappa Q\cos(\beta-\phi)} d\beta \\ &= \begin{cases} 2\pi[J_0(\kappa Q) - J_2(\kappa Q)] & \text{for } (j,k) = (1,1) \\ 2\pi[J_0(\kappa Q) + J_2(\kappa Q)] & \text{for } (j,k) = (2,2) \\ 2\pi J_0(\kappa Q) & \text{for} (j,k) = (3,3) \\ 2\pi i J_1(\kappa Q) & \text{for } (j,k) = (1,3) \text{ and } (3,1) \\ 0 & \text{otherwise} \end{cases} \end{aligned} \tag{S48}$$

Noting that these expressions are independent of ϕ we can similarly evaluate the integral over ϕ yielding

$$\mathbb{I}_{jk}^{\beta}(\mathbf{P}-\mathbf{Q}, z_1, z_2) = (2\pi)^2 \int_0^\infty \frac{\kappa^3 B_{jk}(\kappa Q) e^{-\kappa\mathscr{Z}}}{(k_{\mathrm{SPP}}^2 - \kappa^2)(k_{\mathrm{SPP}}^2 - \kappa^2)^*} \mathbb{N}(\kappa P, \theta) d\kappa \tag{S49}$$

where

$$\mathbb{N}(\kappa P,\theta)=\begin{bmatrix}\frac{1}{2}|\alpha|^2[J_0(\kappa P)-J_2(\kappa P)\cos 2\theta] & -\frac{1}{2}|\alpha|^2 J_2(\kappa P)\sin 2\theta & \alpha^* J_1(\kappa P)\cos\theta\\ -\frac{1}{2}|\alpha|^2 J_2(\kappa P)\sin 2\theta & \frac{1}{2}|\alpha|^2[J_0(\kappa P)+J_2(\kappa P)\cos 2\theta] & \alpha^* J_1(\kappa P)\sin\theta\\ -\alpha J_1(\kappa P)\cos\theta & -\alpha J_1(\kappa P)\sin\theta & J_0(\kappa P)\end{bmatrix}. \tag{S50}$$

Now defining the auxiliary matrix

$$\mathbb{V}_n(P,Q,\theta,z_1,z_2)=\int_0^\infty \frac{\kappa^3 J_n(\kappa Q)e^{-\kappa\mathscr{Z}}}{(k_{\text{SPP}}^2-\kappa^2)(k_{\text{SPP}}^2-\kappa^2)^*}\mathbb{N}(\kappa P,\theta)d\kappa \tag{S51}$$

we have

$$\mathbb{I}_{jk}^{\beta}(\mathbf{P}-\mathbf{Q})=(2\pi)^2[\delta_{jk}\{\mathbb{V}_0+(\delta_{j2}-\delta_{j1})\mathbb{V}_2\}+i(\delta_{j1}\delta_{k3}+\delta_{j3}\delta_{k1})\mathbb{V}_1] \tag{S52}$$

and hence

$$\mathbb{W}(\mathbf{r}_1,\mathbf{r}_2)=\frac{(2\pi)^4\omega^2\mu_0^2}{|K_0|^2}\sum_{j,k=1}^{3} m_{jk}\int_0^\infty \Big[\delta_{jk}\{\mathbb{V}_0(P,Q,\theta,z_1,z_2)+(\delta_{j2}-\delta_{j1})\mathbb{V}_2(P,Q,\theta,z_1,z_2)\} + i(\delta_{j1}\delta_{k3}+\delta_{j3}\delta_{k1})\mathbb{V}_1(P,Q,\theta,z_1,z_2)\Big]Q w_{jk}(Q)dQ. \tag{S53}$$

Eq. (S53) can however be simplified further by considering the assumption $w_{jk}(\mathbf{Q})=w_{jk}(Q)$ further. In particular, we require this angular independence to hold regardless of the two points $\mathbf{r}_1$ and $\mathbf{r}_2$ under consideration, i.e. for general $\mathbf{Q}$. Satisfying this requirement, necessitates that $\mathbb{w}_J$ must be invariant under rotations around the z axis, and therefore $w_{11}=w_{22}$, $w_{12}=-w_{21}$, $w_{13}=w_{31}=w_{23}=w_{32}=0$. Rewriting Eq. (S53) in the form

$$\mathbb{W}(\mathbf{P},z_1,z_2)=\frac{(2\pi)^4\omega^2\mu_0^2}{|K_0|^2}\sum_{k=0}^{2}\int_0^\infty c_k(Q)\mathbb{V}_k(P,Q,\theta,z_1,z_2)dQ \tag{S54}$$

where, recalling $m_{11}=m_{22}$,

$$c_0(Q)=\sum_{j,k=1}^{3} m_{jk}Q\delta_{ij}w_{jk}(Q)\equiv Q\{|\alpha|^2 w_{11}(Q)+w_{33}(Q)\} \tag{S55}$$

$$c_1(Q)=i\sum_{j,k=1}^{3} m_{jk}Q(\delta_{j1}\delta_{k3}+\delta_{j3}\delta_{k1})w_{jk}(Q)\equiv 0 \tag{S56}$$

$$c_2(Q)=\sum_{j,k=1}^{3} m_{jk}Q\delta_{jk}(\delta_{j2}-\delta_{j1})w_{jk}(Q)\equiv 0 \tag{S57}$$

and δ_{jk} is the Kronecker delta, we find the simplified form for the correlation matrix as

$$\mathbb{W}(\mathbf{P},z_1,z_2)=\frac{(2\pi)^4\omega^2\mu_0^2}{|K_0|^2}\int_0^\infty Q\{|\alpha|^2 w_{11}(Q)+w_{33}(Q)\}\mathbb{V}_0(P,Q,\theta,z_1,z_2)dQ. \tag{S58}$$

Our final task thus remains to find an expression for $\mathbb{V}_0$ (we can also find expressions for $\mathbb{V}_n$ without great effort and thus we keep things general for the sake of interest). In particular we must evaluate the integrals

$$L_{nm}=\int_0^\infty \frac{\kappa^3 J_n(\kappa Q)J_m(\kappa P)e^{-\kappa\mathscr{Z}}}{(k_{\text{SPP}}^2-\kappa^2)(k_{\text{SPP}}^2-\kappa^2)^*}d\kappa \tag{S59}$$

which make up the individual terms in $\mathbb{V}_n$ viz.

$$\mathbb{V}_n(P,Q,\theta,z_1,z_2)=\begin{bmatrix}\frac{1}{2}|\alpha|^2[L_{n0}(P,Q)-L_{n2}(P,Q)\cos 2\theta] & -\frac{1}{2}|\alpha|^2 L_{n2}(P,Q)\sin 2\theta & \alpha^* L_{n1}(P,Q)\cos\theta\\ -\frac{1}{2}|\alpha|^2 L_{n2}(P,Q)\sin 2\theta & \frac{1}{2}|\alpha|^2[L_{n0}(P,Q)+L_{n2}(P,Q)\cos 2\theta] & \alpha^* L_{n1}(P,Q)\sin\theta\\ -\alpha L_{n1}(P,Q)\cos\theta & -\alpha L_{n1}(P,Q)\sin\theta & L_{n0}(P,Q)\end{bmatrix}. \tag{S60}$$

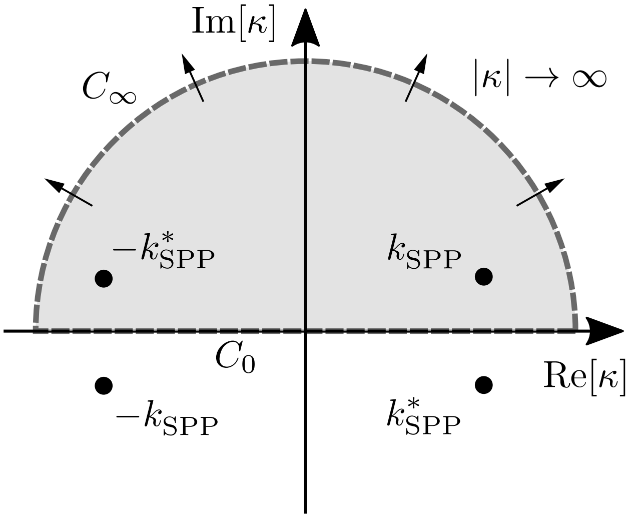

Figure S1. Integration contour on the complex κ plane used to evaluate L_{mn} and positions of the SPP related poles.

We begin by first noting that $J_m(\kappa P) = [H_m^{(1)}(\kappa P) + H_m^{(2)}(\kappa P)]/2$ where $H_m^{(1,2)}$ are the Hankel functions of the first and second kind of order m. This substitution however introduces a pole at $\kappa = 0$ that must be excluded, such that we consider

$$L_{nm} = \frac{1}{2}\lim_{\delta\to 0}\left[\int_\delta^\infty \frac{\kappa^3 J_n(\kappa Q)H_m^{(1)}(\kappa P)e^{-\kappa\mathscr{Z}}}{(k_{\rm SPP}^2-\kappa^2)(k_{\rm SPP}^2-\kappa^2)^*}d\kappa + \int_\delta^\infty \frac{\kappa^3 J_n(\kappa Q)H_m^{(2)}(\kappa P)e^{-\kappa\mathscr{Z}}}{(k_{\rm SPP}^2-\kappa^2)(k_{\rm SPP}^2-\kappa^2)^*}d\kappa\right]. \tag{S61}$$

Now letting $\kappa = e^{-i\pi}\kappa'$ and using the reflection formulae $H_m^{(2)}(e^{-i\pi}z) = -e^{-m\pi i}H_m^{(1)}(z)$ and $J_n(e^{-i\pi}z) = e^{-n\pi i}J_n(z)$[5] we have

$$L_{nm} = \frac{1}{2}\lim_{\delta\to 0}\left[\int_\delta^\infty \frac{\kappa^3 J_n(\kappa Q)H_m^{(1)}(\kappa P)e^{-\kappa\mathscr{Z}}}{(k_{\rm SPP}^2-\kappa^2)(k_{\rm SPP}^2-\kappa^2)^*}d\kappa + (-1)^{n+m}\int_{-\infty}^{-\delta} \frac{\kappa'^3 J_n(\kappa' Q)H_m^{(1)}(\kappa' P)e^{\kappa'\mathscr{Z}}}{(k_{\rm SPP}^2-\kappa'^2)(k_{\rm SPP}^2-\kappa'^2)^*}d\kappa'\right]. \tag{S62}$$

The two integrals can be combined into a single integral according to

$$L_{nm} = \frac{1}{2}\mathrm{PV}\left[\int_{-\infty}^{\infty} g(\kappa)d\kappa\right] = \frac{1}{2}\left[\int_{C_0} g(\kappa)d\kappa - \mathop{\mathrm{Res}}_{\kappa=0}[g(\kappa)]\right] \tag{S63}$$

where PV denotes the principal value, Res denotes the complex residue, the integration contour C_0 is shown in Figure S1 and $g(\kappa)$ is an analytic function in the upper half plane given by

$$g(\kappa) = (\mathrm{sign}[\mathrm{Re}(\kappa)])^{n+m}\frac{\kappa^3 J_n(\kappa Q)H_m^{(1)}(\kappa P)}{(k_{\rm SPP}^2-\kappa^2)(k_{\rm SPP}^2-\kappa^2)^*}\exp[-\kappa\sigma(\kappa)\mathscr{Z}] \tag{S64}$$

with $\sigma = \mathrm{sign}[\mathrm{Re}(\kappa)]$. To examine the residue at $\kappa = 0$ we can consider the small κ behaviour of $g(\kappa)$ using the small argument expansions[5]

$$J_n(z) \sim \left(\frac{z}{2}\right)^n/\Gamma(n+1) \tag{S65}$$

$$H_n^{(1)}(z) \sim -\frac{i}{\pi}\Gamma(n)\left(\frac{z}{2}\right)^{-n}, \tag{S66}$$

where $\Gamma(n)$ is the Gamma function. Specifically

$$\mathop{\mathrm{Res}}_{\kappa=0}[g(\kappa)] = \lim_{\kappa\to 0}[\kappa g(\kappa)] = \lim_{\kappa\to 0}\left[\kappa^{n-m}\left(\frac{\kappa^4}{|k_{\rm SPP}|^4}+\dots\right)\right] \tag{S67}$$

which tends to zero for $m-n \geq 3$ cases considered in this work.

With a view to extending the integration contour C_0 we consider the behaviour of $J_n(\kappa Q)H_m^{(1)}(\kappa P)$ for $|\kappa| \to \infty$. The asymptotic formulae

$$J_n(z) = \sqrt{\frac{2}{\pi z}}\cos(z - n\pi/2 - \pi/4) + \cdots \tag{S68}$$

$$H_n^{(1)}(z) = \sqrt{\frac{2}{\pi z}}\exp[i(z - n\pi/2 - \pi/4)] + \cdots \tag{S69}$$

give

$$J_n(\kappa Q)H_m^{(1)}(\kappa P) \to \frac{2}{\pi\kappa\sqrt{PQ}}\left[e^{i\kappa(P+Q)}e^{-i\pi(n+m+1)/2} + e^{i\kappa(P-Q)}e^{i\pi(n-m)/2}\right]. \tag{S70}$$

Splitting $\kappa = \kappa_r + i\kappa_i$ into its real and imaginary parts, we see that the two position dependent exponents scale as $e^{i\kappa(P\pm Q)} \sim e^{-\kappa_i(P\pm Q)}$, both of which decay for $\kappa_i > 0$, $|\kappa| \to \infty$ if $P > Q$. Assuming this latter condition to hold true, the integration contour C_0 in Eq. (S63) can be extended to include the semicircular contour C_∞ (as shown in Figure S1) where the radius of C_∞ is taken to infinity, since the value of the integrand, $g(\kappa)$, is zero along this contour. Accordingly the integration path is now a closed loop and noting Jordan's lemma, the integral can be evaluated using the residual theorem. The function $g(\kappa)$ has four simple poles at $\kappa = \pm k_{\mathrm{SPP}}$ and $\pm k_{\mathrm{SPP}}^*$, only two of which lie within the integration contour namely $\kappa_1 = k_{\mathrm{SPP}}$ and $\kappa_2 = -k_{\mathrm{SPP}}^*$. Accordingly

$$L_{nm} = \pi i\left[\operatorname*{Res}_{\kappa=k_{\mathrm{SPP}}}[g(\kappa)] + \operatorname*{Res}_{\kappa=-k_{\mathrm{SPP}}^*}[g(\kappa)]\right] \tag{S71}$$

$$= \pi i\left[\lim_{\kappa\to k_{\mathrm{SPP}}}[(\kappa - k_{\mathrm{SPP}})g(\kappa)] + \lim_{\kappa\to -k_{\mathrm{SPP}}^*}[(\kappa + k_{\mathrm{SPP}}^*)g(\kappa)]\right]. \tag{S72}$$

Evaluating the limits gives $L_{nm}(P,Q) = l_{nm}(P,Q,k_{\mathrm{SPP}}) + l_{nm}(P,Q,-k_{\mathrm{SPP}}^*)$ where

$$l_{nm}(P,Q,\kappa) = \sigma(\kappa)^{n+m}\frac{\pi}{4}\frac{\kappa^2}{\mathrm{Im}[\kappa^2]}J_n(\kappa Q)H_m^{(1)}(\kappa P)\exp[-\kappa\sigma(\kappa)\mathscr{Z}]. \tag{S73}$$

For the case $P < Q$, instead of initially expanding $J_m(\kappa P)$ in terms of the Hankel functions in Eq. (S61), we make the alternative substitution $J_n(\kappa Q) = [H_n^{(1)}(\kappa Q) + H_n^{(2)}(\kappa Q)]/2$ which upon following the same logic as above gives

$$l_{nm}(P,Q,\kappa) = \Lambda_{nm}(\kappa)\begin{cases} J_n(\kappa Q)H_m^{(1)}(\kappa P) & \text{for } P > Q \\ H_n^{(1)}(\kappa Q)J_m(\kappa P) & \text{for } P < Q \end{cases} \tag{S74}$$

where $\Lambda_{nm}(\kappa) = \sigma(\kappa)^{n+m}\pi\kappa^2\exp[-\kappa\sigma(\kappa)\mathscr{Z}]/(4\mathrm{Im}[\kappa^2])$.

S2 Degree of polarisation

In this section we evaluate the 2D and 3D degrees of polarisation (DOP) at $\mathbf{P} = \mathbf{0}$ defined by[6,7]

$$(\mathscr{D}_{2\mathrm{D}})^2 = 2\left\{\frac{\|\mathbb{W}(\mathbf{0},z_1,z_2)\|_F^2}{\mathrm{tr}[\mathbb{W}(\mathbf{0},z_1,z_2)]^2} - \frac{1}{2}\right\}, \tag{S75}$$

and

$$(\mathscr{D}_{3\mathrm{D}})^2 = \frac{3}{2}\left\{\frac{\|\mathbb{W}^2(\mathbf{0},z_1,z_2)\|_F^2}{\mathrm{tr}[\mathbb{W}(\mathbf{0},z_1,z_2)]^2} - \frac{1}{3}\right\}, \tag{S76}$$

respectively where $\|\cdots\|_F$ denotes the Frobenius norm. We thus must first evaluate $\mathbb{W}(\mathbf{0},z_1,z_2)$. Using Eqs. (S60) and (S74) at $P = 0$ we have $l_{nm}(0,Q,\kappa) = \Lambda_{nm}(\kappa)H_n^{(1)}(\kappa Q)\delta_{m0}$ such that

$$\mathbb{V}_n(0,Q,\theta,z_1,z_2) = \begin{bmatrix} \frac{1}{2}|\alpha|^2 & 0 & 0 \\ 0 & \frac{1}{2}|\alpha|^2 & 0 \\ 0 & 0 & 1 \end{bmatrix} L_{n0} \triangleq \mathbb{A}L_{n0} \tag{S77}$$

and

$$\mathbb{W}(\mathbf{P}=\mathbf{0},z_1,z_2)=\mathbb{A}\left[\frac{(2\pi)^4\omega^2\mu_0^2|\alpha|^2}{|K_0|^2}\int_0^\infty Q w_{11}(Q)L_{00}(0,Q)dQ\right]=W_0\mathbb{A}, \tag{S78}$$

where W_0 is a constant found by evaluating the integral. Substituting this expression into Eq. (S76) directly gives

$$\mathscr{D}_{3\mathrm{D}}=\frac{|\alpha|^2-2}{2(1+|\alpha|^2)}. \tag{S79}$$

For the 2D case describing correlations between in-plane field components we consider the reduced correlation matrix

$$\mathbb{W}_{\parallel}(\mathbf{P}=0,z_1,z_2)=W_0\left[\begin{array}{cc}\frac{1}{2}|\alpha|^2 & 0\\ 0 & \frac{1}{2}|\alpha|^2\end{array}\right] \tag{S80}$$

yielding $\mathscr{D}_{2\mathrm{D}}^{\parallel}=0$, whilst to describe the 2D DOP between in and out-of-plane field components we consider

$$\mathbb{W}_{\perp}(\mathbf{P}=0,z_1,z_2)=W_0\left[\begin{array}{cc}\frac{1}{2}|\alpha|^2 & 0\\ 0 & 1\end{array}\right] \tag{S81}$$

whereby

$$\mathscr{D}_{2\mathrm{D}}^{\perp}=\frac{\sqrt{1-2|\alpha|^2}}{1+|\alpha|^2}. \tag{S82}$$

S3 Degree of coherence

The degree of coherence of a 3D field is defined as $\mu(\mathbf{P},z_1,z_2)=\mathrm{tr}[\mathbb{W}(\mathbf{P},z_1,z_2)]/\mathrm{tr}[\mathbb{W}(\mathbf{0},0,0)]$. Noting that $\mathrm{tr}[\mathbb{V}_n]=(|\alpha|^2+1)L_{n0}$ taking the trace of Eq. (S58) yields

$$\mathrm{tr}[\mathbb{W}(\mathbf{P},z_1,z_2)]=\frac{(2\pi)^4\omega^2\mu_0^2}{|K_0|^2}(|\alpha|^2+1)\int_0^\infty Q\{|\alpha|^2w_{11}(Q)+w_{33}(Q)\}L_{00}(P,Q,z_1,z_2)dQ. \tag{S83}$$

Letting $T_0=(2\pi)^4\omega^2\mu_0^2(|\alpha|^2+1)/|K_0|^2$ and $\mathscr{W}(Q)=|\alpha|^2w_{11}(Q)+w_{33}(Q)$, we can split the integration domain such that

$$\mathrm{tr}[\mathbb{W}(\mathbf{P},z_1,z_2)]=T_0\left[\int_0^P Q\mathscr{W}(Q)L_{00}(P,Q,z_1,z_2)dQ+\int_P^\infty Q\mathscr{W}(Q)L_{00}(P,Q,z_1,z_2)dQ\right]. \tag{S84}$$

All values of Q in the integration domain of the first term are such that $Q<P$ and the L_{k0} factors are given by the first expression in Eq. (S74), whereas the converse is true for the second integral in Eq. (S84). Hence we can write

$$\mathrm{tr}[\mathbb{W}(\mathbf{P},z_1,z_2)]=T_0\sum_{p=1}^{2}\Lambda_{00}(\kappa_p)\left[H_0^{(1)}(\kappa_pP)\int_0^P Q\mathscr{W}(Q)J_0(\kappa_pQ)dQ+J_0(\kappa_pP)\int_P^\infty Q\mathscr{W}(Q)H_0^{(1)}(\kappa_pQ)dQ\right]. \tag{S85}$$

Noting that $\Lambda_{k0}(\kappa_2)=-(-1)^k\Gamma\Lambda_{00}(\kappa_1)$, where $\Gamma=(\kappa_2/\kappa_1)^2\exp[(\kappa_1+\kappa_2)\mathscr{Z}]$ we can further write

$$\mathrm{tr}[\mathbb{W}(\mathbf{P},z_1,z_2)]=T_0\Lambda_{00}(\kappa_1)\sum_{p=1}^{2}r_p\left[H_0^{(1)}(\kappa_pP)\int_0^P Q\mathscr{W}(Q)J_0(\kappa_pQ)dQ+J_0(\kappa_pP)\int_P^\infty Q\mathscr{W}(Q)H_0^{(1)}(\kappa_pQ)dQ\right] \tag{S86}$$

where $r_1=1$ and $r_2=-\Gamma$. Hence

$$\mu(\mathbf{P},z_1,z_2)=e^{-\kappa_1\mathscr{Z}}\frac{\sum_{p=1}^2 r_p\left[H_0^{(1)}(\kappa_pP)\int_0^P Q\mathscr{W}(Q)J_0(\kappa_pQ)dQ+J_0(\kappa_pP)\int_P^\infty Q\mathscr{W}(Q)H_0^{(1)}(\kappa_pQ)dQ\right]}{\sum_{p=1}^2 r_p\int_0^\infty Q\mathscr{W}(Q)H_0^{(1)}(\kappa_pQ)dQ}. \tag{S87}$$

Eq. (S87) gives a general expression for the DOC which is dependent on the surface and source correlation functions through $w_{11}=w_{22}$ and w_{33}, however, at this point we make a further approximation. Specifically, we split $\kappa_1=k_{\mathrm{SPP}}$ into its real and imaginary parts viz. $\kappa_1=\kappa_{1r}+i\kappa_{1i}$ and note that for typical SPPs $\kappa_{1i}\ll\kappa_{1r}$. We wish to approximate the integral terms in

Eq. (S87) and thus we expand the arguments of the Bessel and Hankel functions as $\kappa_{1r}Q + i\kappa_{1i}Q$. For $\kappa_{1i}Q \ll 1$ (or equivalently $Q \ll 2L$ where $L = 1/(2\text{Im}[k_{\text{SPP}}])$ is the SPP attenuation length) we can perform a series expansion about $\kappa_{1i} = 0$ to first order. Doing this yields an expression of the same form as Eq. (S87) albeit with the replacements $J_0(\kappa_p Q) \to J_0(\kappa_{rp}Q)$ and similarly for the $H_0(\kappa_p Q)$ terms, where $\kappa_{r2} = -\kappa_{r1}$. Using the reflection formulae for the Bessel and Hankel functions[5] we have $J_0(\kappa_{2r}Q) = J_0(\kappa_{1r}Q)$, $H_0^{(1)}(\kappa_{2r}Q) = -H_0^{(2)}(\kappa_{1r}Q)$ yielding

$$\mu(\mathbf{P}, z_1, z_2) \approx \frac{1}{2} e^{-\kappa_1 \mathscr{Z}} \left\{ \left[H_0^{(1)}(\kappa_1 P) - H_0^{(1)}(\kappa_2 P) \right] f_J^P(P) \right.$$
$$\left. + \left[J_0(\kappa_1 P) + J_0(\kappa_2 P) \right] f_J^\infty(P) + i \left[J_0(\kappa_1 P) - J_0(\kappa_2 P) \right] f_Y^\infty(P) \right\} \tag{S88}$$

where

$$f_J^P(P) = \frac{\int_0^P Q \mathscr{W}(Q) J_0(\kappa_{1r}Q) dQ}{\int_0^\infty Q \mathscr{W}(Q) J_0(\kappa_{1r}Q) dQ} \tag{S89}$$
$$f_J^\infty(P) = \frac{\int_P^\infty Q \mathscr{W}(Q) J_0(\kappa_{1r}Q) dQ}{\int_0^\infty Q \mathscr{W}(Q) J_0(\kappa_{1r}Q) dQ} = 1 - f_J^P(P) \tag{S90}$$
$$f_Y^\infty(P) = \frac{\int_P^\infty Q \mathscr{W}(Q) Y_0(\kappa_{1r}Q) dQ}{\int_0^\infty Q \mathscr{W}(Q) J_0(\kappa_{1r}Q) dQ} \tag{S91}$$

Upon making a number of further approximations we can simplify Eq. (S87) for two cases, namely that of (i) a loss-free metal such that ε_2 is purely real and negative, and (ii) a narrow correlation function w_{jk}. We shall consider both cases now in turn.

S3.1 Loss-free case

If we assume that the electric permittivity of the metal is purely real and negative then consequently k_{SPP} and α are purely real (the dielectric is also assumed lossless) then $\kappa_1 = -\kappa_2$. Accordingly Eq. (S88) is exact. We can however simplify the terms in square brackets appearing in Eq. (S88) further by again recalling the reflection formulae for the Bessel and Hankel functions. Specifically we have

$$H_0^{(1)}(\kappa_1 P) - H_0^{(1)}(\kappa_2 P) = H_0^{(1)}(\kappa_{1r} P) + H_0^{(2)}(\kappa_{1r} P) = 2J_0(\kappa_{1r} P) \tag{S92}$$
$$J_0(\kappa_1 P) + J_0(\kappa_2 P) = J_0(\kappa_{1r} P) + J_0(\kappa_{1r} P) = 2J_0(\kappa_{1r} P) \tag{S93}$$
$$J_0(\kappa_1 P) - J_0(\kappa_2 P) = J_0(\kappa_{1r} P) - J_0(\kappa_{1r} P) = 0 \tag{S94}$$

Substituting these results into Eq. (S88) and using Eq. (S90) it follows that the DOC reduces to the universal form

$$\mu(\mathbf{P}, z_1, z_2) = J_0(\kappa_1 P) \tag{S95}$$

irrespective of the form of the correlation matrix $\mathbb{w}_J$ as has been previously shown for scalar 2D waves[8].

S3.2 Narrow correlation function

Typically the correlation functions $w_{11}(Q) = w_{22}(Q)$ and $w_{33}(Q)$ fall-off to negligible values as Q increases such that we can define a characteristic length Q_0 above which $w_{11}(Q) \approx 0$. When considering points $\mathbf{P}$ such that $P > Q_0$ we can note that the second term in both Eq. (S87) is negligible such that

$$\mu(\mathbf{P}, z_1, z_2) = e^{-\kappa_1 \mathscr{Z}} \frac{\sum_{p=1}^2 r_p H_0^{(1)}(\kappa_p P) \int_0^P Q \mathscr{W}(Q) J_0(\kappa_p Q) dQ}{\sum_{p=1}^2 r_p \int_0^\infty Q \mathscr{W}(Q) H_0^{(1)}(\kappa_p Q) dQ} \tag{S96}$$
$$\approx \frac{1}{2} e^{-\kappa_1 \mathscr{Z}} \left[H_0^{(1)}(\kappa_1 P) - H_0^{(1)}(\kappa_2 P) \right] f_J^P(P). \tag{S97}$$

Since, however, for $Q > Q_0$ we have assumed that the $w_{11}(Q) \approx w_{33}(Q) \approx 0$, the integration limits in Eq. (S89) can both be replaced by $\int_0^{Q_0} \cdots dQ$ without affecting the result. Accordingly $f_J^P = 1$ for all $P > Q_0$ and the DOC reduces to the form

$$\mu(\mathbf{P}, z_1, z_2) \approx \frac{1}{2} e^{-\kappa_1 \mathscr{Z}} \left[H_0^{(1)}(\kappa_1 P) - H_0^{(1)}(\kappa_2 P) \right]. \tag{S98}$$

It is worthwhile to note at this point that the DOC for points separated by a distance greater than the characteristic correlation length Q_0 is described by a form that is independent of the precise nature of the source and surface correlation functions. This

statement is however only true for $P \gtrsim Q_0$, meaning when considering more closely spaced points the DOC between these positions exhibits non-universal behaviour i.e. the precise functional form of μ depends on $\mathbb{W}_J$. Nevertheless, for very narrow correlation functions (i.e. $Q_0 \to 0$) the DOC is given by Eq. (S98) for all $P \neq 0$. By construction we note that $\mu(\mathbf{0},0,0) = 1$. Importantly, Eq. (S98) does not hold in the large loss limit as can be seen by studying the limiting behaviour as $P \to 0$. In particular

$$\lim_{P\to 0} \frac{1}{2}\left[H_0^{(1)}(\kappa_1 P) - H_0^{(1)}(\kappa_2 P)\right] = \frac{i}{\pi}[\log(\kappa_1) - \log(\kappa_2)] \tag{S99}$$

which does not equal unity for $k_{1i} > 0$.

References

1. Farias, G. A. & Maradudin, A. A. Surface plasmons on a randomly rough surface. *Phys. Rev. B* **28**, 5675–5687, DOI: 10.1103/PhysRevB.28.5675 (1983).

2. Søndergaard, T. & Bozhevolnyi, S. I. Surface plasmon polariton scattering by a small particle placed near a metal surface: An analytical study. *Phys. Rev. B* **69**, 045422, DOI: 10.1103/PhysRevB.69.045422 (2004).

3. Tomas, M. S. Green function for multilayers: light scattering in planar cavities. *Phys. Rev. A* **51**, 2545–2559 (1995).

4. Stratton, J. A. *Electromagnetic Theory* (McGraw-Hill, New York, 1941), 1 edn.

5. Abramowitz, M. & Stegun, I. A. *Handbook of Mathematical Functions* (Dover Publications, New York, 1972).

6. Setälä, T., Shevchenko, A., Kaivola, M. & Friberg, A. T. Degree of polarization for optical near fields. *Phys. Rev. E* **66**, 016615, DOI: 10.1103/PhysRevE.66.016615 (2002).

7. Tervo, J., Setälä, T. & Friberg, A. T. Degree of coherence for electromagnetic fields. *Opt. Express* **11**, 1137–1143 (2003).

8. Ponomarenko, S. A. & Wolf, E. Universal structure of field correlations within a fluctuating medium. *Phys. Rev. E* **65**, 016602, DOI: 10.1103/PhysRevE.65.016602 (2002).
